# Issues involved in the quantitative 3D imaging of proton doses using optical CT and chemical dosimeters

**DOI:** 10.1088/0031-9155/60/2/709

**Published:** 2015-01-02

**Authors:** Simon Doran, Tina Gorjiara, Andrzej Kacperek, John Adamovics, Zdenka Kuncic, Clive Baldock

**Affiliations:** 1 CRUK Cancer Imaging Centre, Institute of Cancer Research, London, UK; 2 Department of Physics, University of Surrey, Guildford, Surrey, UK; 3 Royal Prince Alfred Hospital, Camperdown, NSW 2050, Australia; 4 Douglas Cyclotron, Clatterbridge Cancer Centre, Wirral, UK; 5 Department of Chemistry and Biology, Rider University, Lawrenceville, NJ 08648, USA; 6 Institute of Medical Physics, School of Physics, University of Sydney, NSW 2006, Australia; Simon.Doran@icr.ac.uk

**Keywords:** optical CT, proton therapy, PRESAGE

## Abstract

Dosimetry of proton beams using 3D imaging of chemical dosimeters is complicated by a variation with proton linear energy transfer (LET) of the dose–response (the so-called ‘quenching effect’). Simple theoretical arguments lead to the conclusion that the total absorbed dose from multiple irradiations with different LETs cannot be uniquely determined from post-irradiation imaging measurements on the dosimeter. Thus, a direct inversion of the imaging data is not possible and the proposition is made to use a forward model based on appropriate output from a planning system to predict the 3D response of the dosimeter.

In addition to the quenching effect, it is well known that chemical dosimeters have a non-linear response at high doses. To the best of our knowledge it has not yet been determined how this phenomenon is affected by LET. The implications for dosimetry of a number of potential scenarios are examined.

Dosimeter response as a function of depth (and hence LET) was measured for four samples of the radiochromic plastic PRESAGE^®^, using an optical computed tomography readout and entrance doses of 2.0 Gy, 4.0 Gy, 7.8 Gy and 14.7 Gy, respectively. The dosimeter response was separated into two components, a single-exponential low-LET response and a LET-dependent quenching. For the particular formulation of PRESAGE^®^ used, deviations from linearity of the dosimeter response became significant for doses above approximately 16 Gy.

In a second experiment, three samples were each irradiated with two separate beams of 4 Gy in various different configurations. On the basis of the previous characterizations, two different models were tested for the calculation of the combined quenching effect from two contributions with different LETs. It was concluded that a linear superposition model with separate calculation of the quenching for each irradiation did not match the measured result where two beams overlapped. A second model, which used the concept of an ‘effective dose’ matched the experimental results more closely. An attempt was made to measure directly the quench function for two proton beams as a function of all four variables of interest (two physical doses and two LET values). However, this approach was not successful because of limitations in the response of the scanner.

## 1. Introduction

Proton therapy (Verhey *et al* 1998, Levin *et al* 2005, Smith 2006, Deluca *et al* 2007, Schulz-Ertner and Tsujii 2007) is an advanced radiotherapy technique with the potential for achieving dose distributions superior to those available in x-ray and electron-beam therapy. Protons deposit a significant fraction of their energy very rapidly at the end of their range in a sharp and well defined region, called the Bragg peak. Clinically, with the steep dose fall-off post-Bragg peak, almost no energy is deposited to adjacent organs distal to the target. As a result, an extremely well localized dose delivery to tumours is achievable with considerable reduction in the dose delivered to surrounding healthy tissues compared with more conventional therapies. Proton therapy thus has the potential to introduce a significant shift in treatment prescription, and, in particular, to change the size of the ‘margin’ added when converting the clinical target volume (CTV) to a planning treatment volume (PTV).

However, the extremely rapid fall-off in dose at the end of the proton range, together with the fact that the range of a proton beam is modulated by the density of the tissue through which it passes, make proton therapy a more technically demanding method of treatment. The possibilities of depositing either significantly too much dose or no dose at all are concerning, because they would result in either serious damage to healthy tissue in the first case, or treatment failure and lack of tumour control in the second.

The recommended devices for calibration dosimetry of proton beams (Verhey *et al* 1998, Deluca *et al* 2007) are calorimeters and ionization chambers, but these are not suitable for the high-resolution 3D mapping needed for whole-system commissioning and the verification of dose plans. A volumetric 3D dosimeter with dose measurement accuracy comparable to that of an ionization chamber, and dimensions similar to those of the tumour and adjacent organs at risk, is thus highly desirable (Zeidan *et al* 2010).

But there is a problem: with the exception of calorimeters, ionization chambers and some silicon devices, all types of radiation dosimeter show a dependence of some form or another on the LET of the incident proton radiation (Karger *et al* 2010). In the context of 3D dosimetry, this means that polyacrylamide gels (PAGs) (Gustavsson *et al* 2004, Baldock *et al* 2010), Fricke gels (Bäck *et al* 1999, Schreiner 2004) and PRESAGE^®^ (Adamovics and Maryanski 2006, Al-Nowais *et al* 2009, 2010) all under-record dose at depths near the Bragg peak. This phenomenon is often known as signal ‘quenching’ and, as we show below, is linked to the high-dose saturation that has long been observed in traditional low-LET measurements, particularly for PAG.

Jirasek and Duzenli (2002) observed quenching effects in polymer gel samples and explained their findings using the framework of track structure theory, whilst Gustavsson *et al* (2004) interpreted similar observations in terms of ion-recombination. Zeidan *et al* (2010) presented data showing negligible quenching in high-LET regions, in marked contrast to what had been observed by Gustavsson *et al*. However, whilst this discrepancy may have been due to an improved gel formulation, as claimed, there was also a significant energy difference between the proton beams used. This changes the sharpness of the Bragg peak and the peak-to-entrance dose ratio. Typical values are approximately 5:1 at 60 MeV (Al-Nowais *et al* 2009), 3.3:1 at 133 MeV (Gustavsson *et al* 2004) and 2.2:1 at 250 MeV (Zeidan *et al* 2010). Range straggling affects not only the height and width of the physical Bragg peak (actual dose deposited), but also the spread of LETs of the protons depositing energy within a typical imaging voxel. Thus, if quenching is a function of LET, it is not unreasonable for the magnitude of the observed effect to depend both on the spatial resolution of the imaging technique and on energy, with more significant quenching observed at lower energy, where high LET values in an imaging voxel are less ‘diluted’. This hypothesis would also explain why previous experience of 3D dosimetry using modulated proton beams (‘spread-out Bragg peak’) has demonstrated only minor quenching effects, limited to the distal end of the dose distribution.

As was alluded to above, the problem is not unique to 3D dosimetry: analogous results are also seen using other dosimeters, including radiochromic film (Kirby *et al* 2010), alanine (Herrmann *et al* 2011) and liquid fluorescence dosimeters (Nadrowitz *et al* 2012).

Despite the clear challenge posed by quenching, previous authors have been optimistic about the potential of 3D dosimetry. Zhao *et al* (2012) observed a significant under-response at the Bragg peak, but concluded that this could be overcome by appropriate calibration.

The aim of this study, therefore, was to measure in greater detail the quenching effect and to investigate the proposition that an empirical correction can be performed on optical computed tomography (CT) data to derive a physical dose distribution. As a by-product of this investigation, we report findings that raise some concerns on the quantitative values returned by optical CT dosimetry for samples containing highly absorbing regions.

## 2. Theory

### 2.1. Comparison of ion chamber and imaging measurements

For simplicity, let us suppose that it is possible to represent the LET effect of a proton beam by some form of ‘average’ LET within a given imaging voxel, which we denote by $\mathcal{L}$(***r***). If *D*(***r***) is the physical dose and *I*(***r***) represents an imaging readout, which might be an optical CT image intensity (as used here) or an MRI *R*_2_ value, then we can write

1 \begin{eqnarray*}I~=~I\left(D,~\mathcal{L}\right)=~I\left(D,~{{\mathcal{L}}_{0}}\right)\cdot ~q\left(D,\mathcal{L}\right),\end{eqnarray*}

where $I\left(D,{{\mathcal{L}}_{0}}\right)$ is the low-LET calibration function relating dose to image intensity in the absence of quenching, and the spatial dependence of all the quantities is henceforth suppressed for brevity. $I\left(D,{{\mathcal{L}}_{0}}\right)$ can be measured from a dose–response curve using photon irradiation, or, as here, estimated from the response to low-LET protons at the entrance. *q*, the quenching function, is thus defined as the ratio of the imaging readout measured by the actual 3D dosimetry measurement at a given LET to the readout that *would have occurred* had the same dose been deposited by low LET radiation.

This definition carries with it some subtleties. To see this, we need to consider how to compare the output of the imaging experiment with the reference output from an ionization chamber. Since the units of image intensity and ionization chamber current are not directly interconvertible, we need to normalize both sets of data. It is convenient to do this at the (low-LET) *entrance*, which in our case, will correspond to the shallowest depth *d*_0_ in the sample for which a reliable optical CT measurement is available. The measurement reported in the Results section below will be the optical CT-ionization chamber ratio, defined as:

2 \begin{eqnarray*}R\left(D,\mathcal{L}\right)=\frac{I(d) /I\left({{d}_{0}}\right)}{{{I}_{\text{ic}}}(d) /{{I}_{\text{ic}}}\left({{d}_{0}}\right)}=\frac{I\left(D,\mathcal{L}\right) /I\left({{D}_{0}},{{\mathcal{L}}_{0}}\right)}{{{I}_{\text{ic}}}\left(D,\mathcal{L}\right) /{{I}_{\text{ic}}}\left({{D}_{0}},{{\mathcal{L}}_{0}}\right)},\end{eqnarray*}

where *I*_ic_ represents the signal from the ionization chamber and *d* is the depth in the sample. Note that the ionization chamber reading is assumed to be a secondary or tertiary standard, independent of LET and directly proportional to the dose, so that the denominator of the fraction is just *D*/*D*_0_ (In the case of comparison with a treatment planning system, the denominator is trivially the same as this.)

Manipulating equations (1) and (2), we have:

3 \begin{eqnarray*}q\left(D,\mathcal{L}\right)=\frac{I\left(D,\mathcal{L}\right)}{I\left(D,{{\mathcal{L}}_{0}}\right)}\text{}\text{}=\frac{R\left(D,\mathcal{L}\right)}{\left[\frac{I\left(D,\mathcal{L}\right)}{I\left({{D}_{0}},{{\mathcal{L}}_{0}}\right)}/\frac{D}{{{D}_{0}}}\right]}.\end{eqnarray*}

If our gel, PRESAGE^®^ or other 3D dosimeter responds linearly to dose, then the term in square brackets is unity and so dividing the normalized image by the normalized ion-chamber reading gives the quenching function directly. If, however, the imaging detector inherently has non-linear response for low-LET radiation, as is already known to be the case for polymer gels, then $R\left(D,\mathcal{L}\right)$ needs to be corrected appropriately in order to find the specifically LET-related part of the signal reduction, $q\left(D,\mathcal{L}\right)$.

### 2.2. Inversion of intensity to obtain dose versus forward predictive model of image intensity

It remains to be demonstrated whether the practical impact of quenching will be significant for 3D dosimetry of realistic therapy dose distributions. Nevertheless, it is straightforward to demonstrate mathematically that the very existence of an LET-dependent quenching function immediately implies the theoretical impossibility of deriving relative dose data *directly* from measured optical CT image intensity or MRI *R*_2_ value with no other *a priori* knowledge.

**Figure 1.**
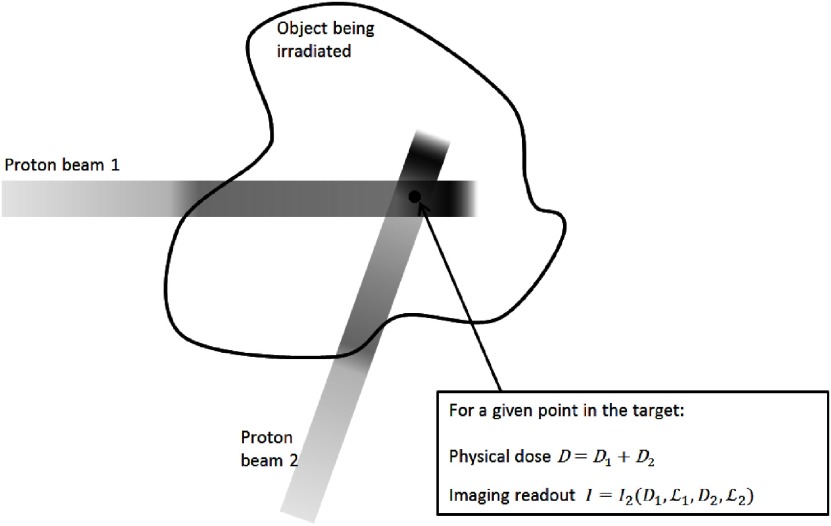
Schematic representation of the two-beam situation considered in theory section of the main text. *D*, *D*_1_ and *D*_2_ represent dose values, whilst the imaging readout might correspond to an optical CT image intensity or an MRI *R*_2_ value. ${{\mathcal{L}}_{1}}$ and ${{\mathcal{L}}_{2}}$ are suitably defined average LET values for the voxel for protons in beams 1 and 2, respectively. *I*_2_ is the image intensity arising from the combination of the two beams with different LETs.

Consider the simple situation in figure 1, where two beams cross having previously passed through different thicknesses of tissue, and suppose that the aim of the dosimetry experiment is to check the dose deposited at the indicated position. This analysis could clearly be extended to a many-beam scenario, and in particular to the volumetric measurement of a spot-scanned, intensity-modulated proton therapy (IMPT) treatment where the aim would be to verify the total physical dose arising from a number of pencil beams that have each been differently range-shifted.

In the case of a dosimeter exhibiting no LET effect (and barring time-dependent effects if the two beams are delivered with a delay between them), the imaging response at the indicated point is simply *I* (*D*_1_ + *D*_2_), which is often linear, but might equally well be some dose-dependent calibration function. The key point is that, given a knowledge of *I*(*D*), we can easily invert the relation (possibly numerically) to determine the total dose deposited, though not, of course, to obtain *D*_1_ and *D*_2_ separately. It is often the case that the constant allowing conversion from image intensity to *absolute* dose is not available, but that normalized maps of *relative* dose are precise and accurate.

By contrast, for the situation where the response does depend on LET, we have

4 \begin{eqnarray*}{{I}_{2}}\left({{D}_{1}},~{{\mathcal{L}}_{1}},~{{D}_{2}},~{{\mathcal{L}}_{2}}\right)~=~I\left({{D}_{1}}+{{D}_{2}},{{\mathcal{L}}_{0}}\right)\cdot ~{{q}_{2}}\left({{D}_{1}},~{{\mathcal{L}}_{1}},~{{D}_{2}},~{{\mathcal{L}}_{2}}\right),\end{eqnarray*}

where we will call *q*_2_ a ‘joint quenching function’. There are several different ways in which *I*_2_ could depend on its parameters and we list them in order of generality.

#### Case 1: $I\left(D,{{\mathcal{L}}_{0}}\right)$ is linear, q is dose-independent.

2.2.1.

This is the simplest scenario and it allows us to make a complete separation of the LET and dose terms:

5 \begin{eqnarray*}{{I}_{2}}=\alpha {{D}_{1}}\cdot q\left({{\mathcal{L}}_{1}}\right)~+\alpha {{D}_{2}}\cdot q\left({{\mathcal{L}}_{2}}\right),\end{eqnarray*}

where $I\left(D,{{\mathcal{L}}_{0}}\right)=~\alpha D$, i.e. *α* is the constant gradient of the dose–response relation. It should be clear from equation (5) that *even if the dose*–*response and quench functions have previously been well measured* to provide a calibration, there is no means of determining the total physical dose unambiguously from an imaging measurement made on the system when all the parameters $\left({{D}_{1}},~{{\mathcal{L}}_{1}},~{{D}_{2}},~{{\mathcal{L}}_{2}}\right)$ on the right-hand side of equation (5) are *a priori* unknown. What should be possible, however, is to work *forwards* from a given treatment plan to *predict* the expected imaging response. In principle, the output of the planning algorithm could include details of both dose and LET, for each beam and at each spatial position. Under these circumstances it might be sufficient to compare the actual imaging results against those predicted and to assume that good agreement means that the dose plan has been delivered successfully. It would be a question of experience gained via simulation to determine whether it is likely that two significantly different treatment plans could lead to similar imaging results.

#### Case 2: $I(D,{{\mathcal{L}}_{0}})$ is linear, q is dose-dependent.

2.2.2.

In principle, a forward model would still be possible and equation (5) would be valid except that it would be necessary to measure $q=q\left(D,\mathcal{L}\right)$ as a function of both parameters.

#### Case 3: $I\left(D,{{\mathcal{L}}_{0}}\right)$ is a non-linear function of dose but is not coupled to the quenching.

2.2.3.

From previous work, for example (De Deene *et al* 2006), it is well known that polymer gels have a non-linear dose response. The saturation dose varies according to composition, but is typically in the region of tens of Gy. As shown below, we find evidence that the batch of PRESAGE^®^ used here also demonstrates non-linear effects. In this case:

6 \begin{eqnarray*}{{I}_{2}}=I\left[{{D}_{1}}\cdot q\left({{\mathcal{L}}_{1}}\right)~+{{D}_{2}}\cdot q\left({{\mathcal{L}}_{2}}\right)\right],\end{eqnarray*}

where *I* is typically a single-exponential recovery curve, and the dosimeter behaves as if it has received an *effective dose* determined by the quenching terms.

A possible scenario where equation (6) might be valid could be the case of quenching due to LET-dependent ion-recombination effects, where decreased response is the result of a lower reaction rate, rather than insufficiency of ‘unconverted targets’ (e.g. gel monomer or leucodye molecules).

#### Case 4: $I(D,{{\mathcal{L}}_{0}})$ is a non-linear function of dose and is coupled to the quenching.

2.2.4.

In this problematic scenario, the two dose contributions are *not* independent. This might, for example be the case for the target theory/track-structure model of quenching (Al-Nowais *et al* 2010, Jirasek and Duzenli 2002, Katz 1978). A localized decrease in unconverted targets, caused by irradiation with dose *D*_1_ applied with LET ${{\mathcal{L}}_{1}}$, could change the reaction of the system to dose *D*_2_ and LET ${{\mathcal{L}}_{2}}$. Further work is needed to develop a sufficient theoretical understanding of the processes involved to find the functional dependence of *I*_2_ on its parameters, in order to develop an appropriate forward model.

The experiments below provide the first exploratory steps towards an empirical determination of the appropriate quenching model to use for the proton PRESAGE^®^ dosimeter.

## 3. Methods

### 3.1. Samples and irradiations

The experiments reported here made use of the ‘proton PRESAGE^®^’ formulation, previously characterized by (Gorjiara *et al* 2012), which has the stoichiometric chemical formula C_304_H_510_N_20_O_71_SBr, and a mass density of 1.11 g cm^−3^ (Heuris Inc, Skillman, NJ). All irradiations used the 60 MeV proton beam at the Douglas Cyclotron, Clatterbridge Centre for Oncology (Wirral, UK). Four cylindrical samples (A–D), of diameter 61 mm and length 90 mm were irradiated from the flat end using an unmodulated, collimated circular beam 10 mm in diameter, with one irradiation per cylinder and respective doses (calculated at the entrance) of A = 2.0, B = 4.0, C = 7.8 and D = 14.7 Gy (to 1 decimal place).

In a second experiment, designed to investigate the effects of overlapping multiple beams, three similar cylindrical samples were irradiated as follows:

Sample E: 4.0 Gy (calculated at the entrance) irradiated end-on, as above, followed by a second irradiation of 4.0 Gy with the sample in an identical position, but with a Perspex sheet of approximate thickness 1 cm inserted in the beam path at the proximal end of the sample. This led to the presence of two overlapping but displaced Bragg peaks.

Sample F: As sample A, but using only a 0.5 cm thickness of Perspex.

Sample G: A first irradiation of 4.0 Gy collimated to a 2  ×  2 cm^2^ square cross-section with the sample end-on to the beam as above, followed by an identical irradiation with the sample rotated by 90° so that the radiation impinged on the curved side of the cylinder. This led to a dose distribution in which every pair of LET values in equation (4) was present within the imaged volume.

### 3.2. Optical CT evaluation

After irradiation, the PRESAGE^®^ dosimeters were transported in a cool-bag and then refrigerated at 5 °C for between two and four days prior to optical CT scanning with an in-house CCD-based device (Krstajic and Doran 2006, 2007). To maximize transmission of light through the PRESAGE^®^ dosimeter and ensure the parallel-beam geometry assumed by the image reconstruction algorithm, a refractive index matching liquid was used, with composition 93.4% 2-ethylhexyl salicylate (Sigma-Aldrich Co. LLC.) and 6.6% 4-methoxycinnamic acid 2-ethylhexyl ester (Sigma-Aldrich Co. LLC). These proportions had previously been determined empirically from the best match found in projections on unirradiated dosimeter regions (data not shown) and corresponded to a calculated refractive index of 1.505. A small quantity of green dye was added to the mixture to match as closely as possible the absorption of unirradiated PRESAGE^®^, a step that allowed us to maximize the useful dynamic range of the projection data. (Our supplier no longer sells the liquid directly, but such products can be easily ordered online, for example from www.fastcolours.co.uk/sample---bestoil-green-bgi-solvent-green-3-ci-61565-200-p.asp.) Raw data for each scan consisted of 400 projection images of the dosimeter, each of matrix size 256  ×  256 pixels, acquired over an angular range of 180°. These were reconstructed into 3D volumes of dimension 256^3^ voxels using standard filtered back-projection. All data processing was performed using IDL (Exelis Visual Information Systems, Boulder, CO).

### 3.3. Data post-processing

Since the Bragg peak covers only a few mm for 60 MeV protons, significant changes in signal occur over a very small number of voxels. Thus, in order to obtain a reliable estimate of the optical CT quenching effect, considerable care was required in processing the data. The individual steps were

**Figure 2.**
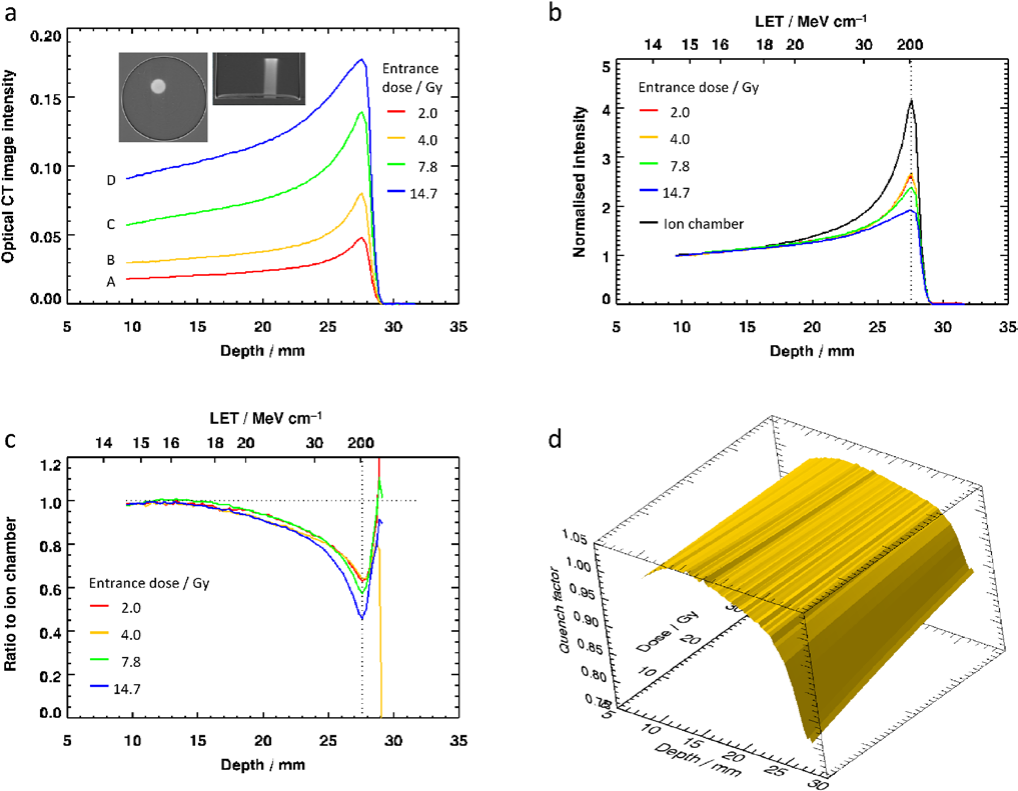
Results of the experiment to measure quenching profiles for different entrance doses: (*a*) profiles from the image data, averaged over a circular region of interest, with inset images of transverse and coronal planes through the highest-dose sample; (*b*) normalized 3D dosimetry profiles, with ion chamber data for comparison; (*c*) ratio of normalized PRESAGE^®^ to normalized ion-chamber reading, defined as $R\left(D,\mathcal{L}\right)$ in the main text; (*d*) the data for $q\left(D,\mathcal{L}\right)$, replotted in terms of depth, since $\mathcal{L}=\mathcal{L}(d)$, and represented as a 2D surface.

• *Averaging over regions-of-interest (ROI)*: The median intensity was calculated from a circular region of radius 3.6 mm (approx. 615 pixels), inscribed within the irradiated region and excluding any penumbra.
• *Subtraction of background*: A 7.5 mm diameter circular background ROI from an empty part of the sample was used on a slice-by-slice basis in the post-irradiation data to determine the background (zero-dose level). An alternative method here would have been to use a pre-scan, but this was not done for three reasons: (i) the data processing (division of two images) required by this step would have introduced additional noise; (ii) other work (data not shown) on this batch of PRESAGE^®^ indicated that some evolution of both irradiated and unirradiated regions in the sample can occur, related to light exposure and thermal history; (iii) the positioning accuracy required for the repeat scan would have increased the complexity of the experiment.
• *Alignment of Bragg peaks*: This compensated for any inconsistency in manual positioning of the samples within the optical CT scanner.
• *Truncation of entrance data*: Quantitative values in the first few mm of the data are strongly affected by image artefacts caused by the interface between PRESAGE^®^ and matching liquid and therefore these regions are not used for entrance-dose calculations. An indication of the magnitude of these effects, together with the influence of the interface of the cylinder with the matching liquid may be seen in figures 2(*a*) and 6(*a*) and (*b*).
• *Conversion of ion chamber data*: An independent calibration of the proton beam in water (supplied by author AK) was recalculated for the equivalent depth in PRESAGE^®^. See Gorjiara *et al* (2012) for discussion.
• *Normalization*: Data were divided by the value at the first point on the truncated profile (9.4 mm depth), as described in section 2.1. Note that ratio to ion chamber *R* and the quenching coefficient *q* are both expected to vary only weakly with distance and dose near this point (see figures 2(*c*) and (*d*)).
• *Ratio to ion chamber readings*: The quantity shown in figure 2(*c*) is $R\left(D,\mathcal{L}\right)$ the ratio of the normalized dose for the PRESAGE^®^ measurements at depth *d* in a sample exposed to an entrance dose of *D* to the normalized ion chamber measurement made at the same depth, as described in the Theory section.
• *Surface fitting:* the variation of the profiles in figure 2(*c*) is a smooth function and thus we can fit an appropriate surface allowing the estimation of the data at an arbitrary intermediate dose.

## 4. Results

### 4.1. Quenching effect

**Figure 3.**
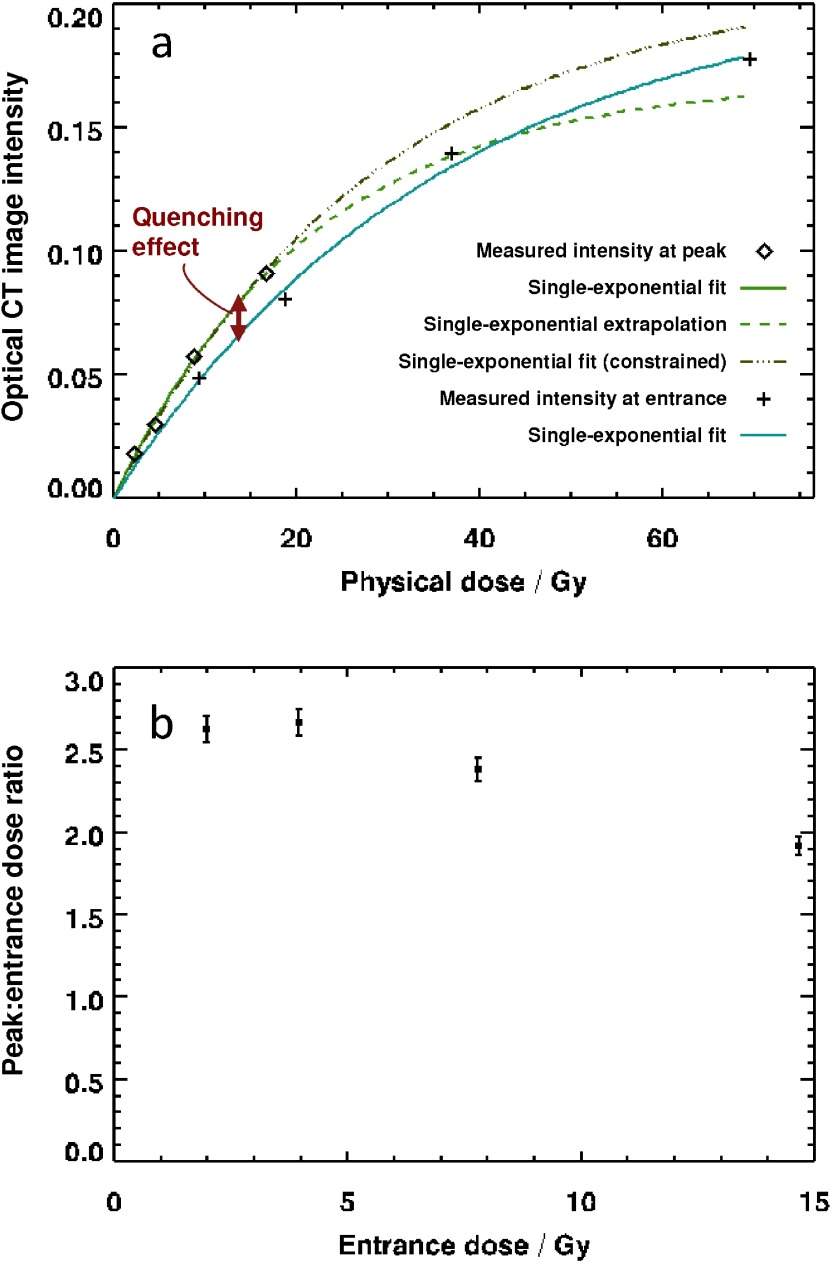
(*a*) Optical CT image intensity data from figure 2(*a*) plotted as a function of dose for the peak and entrance regions of the curve. Fit lines are single-exponential recovery curves, discussed in the main text. The dose values on the horizontal axis have been estimated by multiplying the nominal entrance dose by appropriate ratios obtained from ionization chamber measurements (i.e. correcting for depths of 9.4 and 27.7 mm). Error bars lie within the points and were estimated at 1.5%, corresponding to a small component of random noise (0.3%) and an allowance for sample evolution between scans. (*b*) Measured ratio of optical CT image intensity at the peak and entrance, plotted as a function of dose. Error bars of 3% are the result of dividing two sets of imaging data.

Figures 2 and 3 show the results of the first experiment, carried out to investigate the nature of the quenching as a function of dose. The inset images of figure 2(*a*) demonstrate the quality of the raw data, which is in general high, but the edge artefacts present in the sagittal slice explain why it proved necessary to truncate the start of the profiles, as was also found by (Zhao *et al* 2012). The profiles in figures 2(*a*)–(*d*) show: (*a*) the unnormalized image data; (*b*) the normalized image data; (*c*) $R\left(D,\mathcal{L}\right)$ with separate profiles plotted for each *D*-value acquired, and horizontal axis annotated with both corresponding depth and an approximate LET value; and (*d*) the true quenching function $q\left(D,\mathcal{L}(d)\right)$ plotted as a 2D surface. The LET data used for labelling figures 2(*b*) and (*c*) are indicative only. We approximate the LET in PRESAGE^®^ by that for protons in water (as calculated by the program ‘Energy versus LET versus Range calculator’ version 1.24 by Vladimir Zajic, downloaded from http://tvdg10.phy.bnl.gov/let.html), but with the range scaled by the appropriate ratio of PRESAGE^®^ and water densities. A more detailed examination of the radiological properties of PRESAGE^®^ can be found in (Gorjiara *et al* 2011 2012).

Figure 3(*a*) plots the absolute optical CT image intensities as a function of dose for both the ‘entrance’ data and the ‘peak’ data (i.e. these are the data from figure 2(*a*) at depths of 9.4 and 27.7 mm). The dose values along the *x*-axis are estimates of the true physical dose as calculated from ion-chamber data. The solid lines are an empirical fit to the data with a single-exponential recovery curve, *I* = *I*_0_[1 – exp (*D* / *D*′)], whilst the dotted line is an extrapolation. The extension out to 60 Gy is uncertain, given that the region for which we have entrance-dose data covers only the range 0–14.7 Gy. The lower of the two green fit curves corresponds to the best fit data with a model in which both *I*_0_ and *D*′ are fit parameters. This leads to an apparently unphysical result in which the blue and green curves cross. The upper of the two fit curves to the entrance data corresponds to a model in which *I*_0_ is constrained to the same value as for the peak data, indicating that the blue and green curves would intersect when the dosimeter is completely saturated. It is evident that both of these models fit the measured entrance data equally well. The constant *D*′ is 21.6 Gy (28.6 Gy constrained model) for the entrance data and 36.0 Gy for the peak data.

What figure 3(*a*) demonstrates is that the observed signal reduction at the Bragg peak can be split into two components. Because the physical dose is large at the Bragg peak, there is a reduction in response due to the ‘normal’ (i.e. LET-independent) non-linear dose–response of the samples. But the difference between the green and blue curves shows that there is a second effect, too, which we identify with a genuine quenching phenomenon. The extent of the effect can be established with some confidence up to a physical dose of approximately 20 Gy, but with the data available, there is too much uncertainty in the extrapolation of the entrance dose curve to be able to measure the quenching effect accurately at higher doses.

Arguably, such a high-dose regime is not relevant to clinical practice, but is important for understanding the underlying physics and chemistry of the dose–response. Experimentally, the reasons for not probing this dose region at the time of sample irradiation were (a) the proton facility is not designed to deliver such high doses (70 Gy at the entrance corresponds to over 200 Gy at peak, whereas a typical treatment fraction for uveal melanoma is 13 Gy at peak); (b) were such a dose delivered the samples would have been optically too dense to obtain a CT image over the whole proton range, making absolute calibration of the samples extremely challenging; (c) the sample would be radioactive for some time after exposure, due to the generation of ^11^C, with a half-life of 20 min, via proton activation.

Figure 3(*b*) plots the ratio of the optical CT images intensity at a depth of 27.7 mm to that at 9.4 mm. The corresponding value, as measured by an ionization chamber in water (and appropriately corrected for PRESAGE^®^) is approximately 4.15.

### 4.2. Samples E and F, irradiated with two displaced Bragg peaks

**Figure 4.**
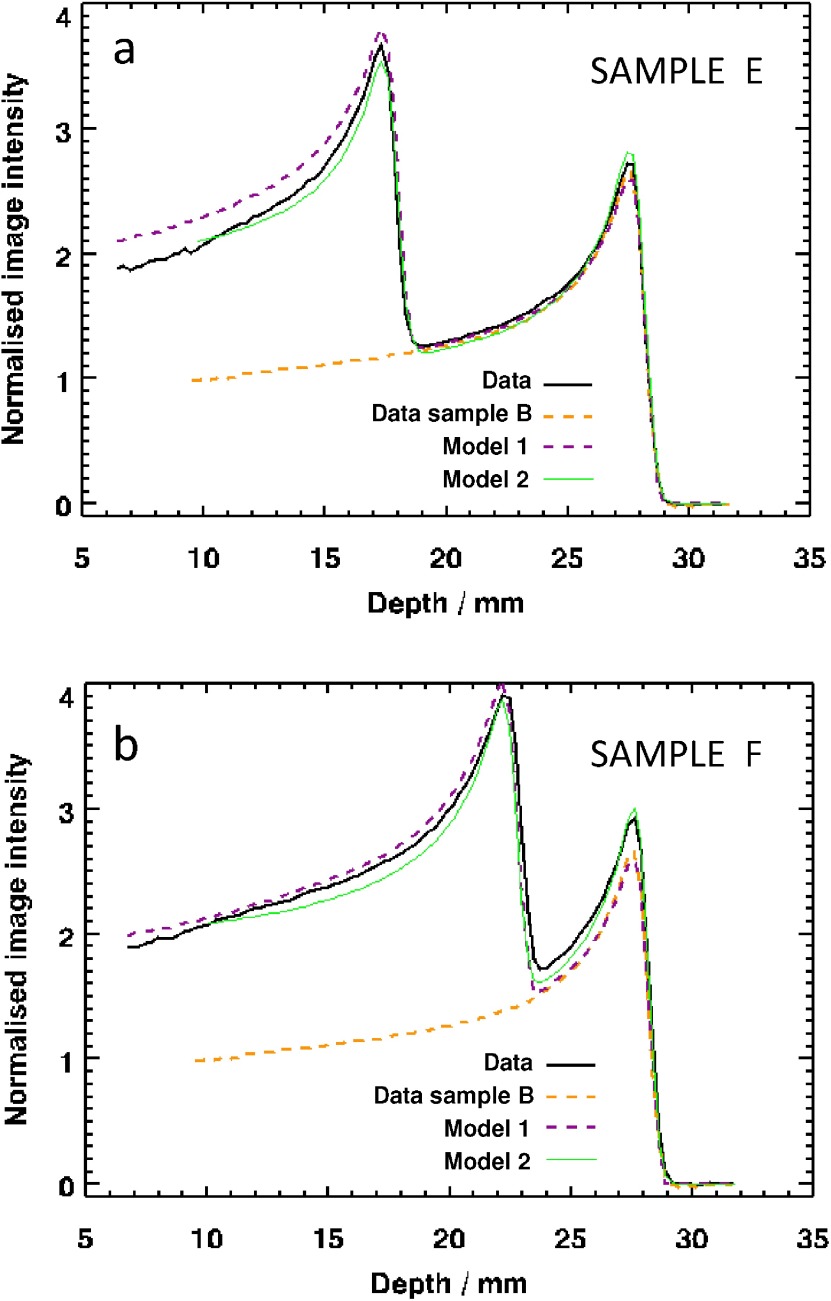
Result of the experiment to irradiate samples with two overlapping Bragg peaks obtained by double irradiation with (*a*) approximately 1 cm, and (*b*) approximately 0.5 cm perspex placed at the proximal end of the sample prior to the second irradiation. The two models correspond to equations (5) and (6), respectively.

Figure 4 shows the results obtained when irradiating samples with two separate Bragg peaks. The aim of the experiment was to test the hypotheses represented by equations (5) and (6). In each case, the two separate irradiations were applied to the samples from the same end and so there was no possibility of performing an internal normalization as with the samples in section 4.1. For the sake of consistency and comparability, it was decided to normalize each of the dose values by the image intensity at a depth of 9.4 mm (i.e. the previously recorded ‘entrance dose’) in the 4 Gy Sample B from section 4.1. The yellow dotted line in figures 4(*a*) and (*b*) is the 4 Gy curve from figure 2(*a*) for reference. It was expected that the portion of the new profiles distal to the non-shifted Bragg peak would match these data. The solid line represents the normalized data for the double-irradiation experiment, whilst the other dotted line is a prediction of the result of the two irradiations using equation (5). The areas of agreement and disagreement of these curves are discussed below.

### 4.3. Sample G, irradiated from the end and the side

**Figure 5.**
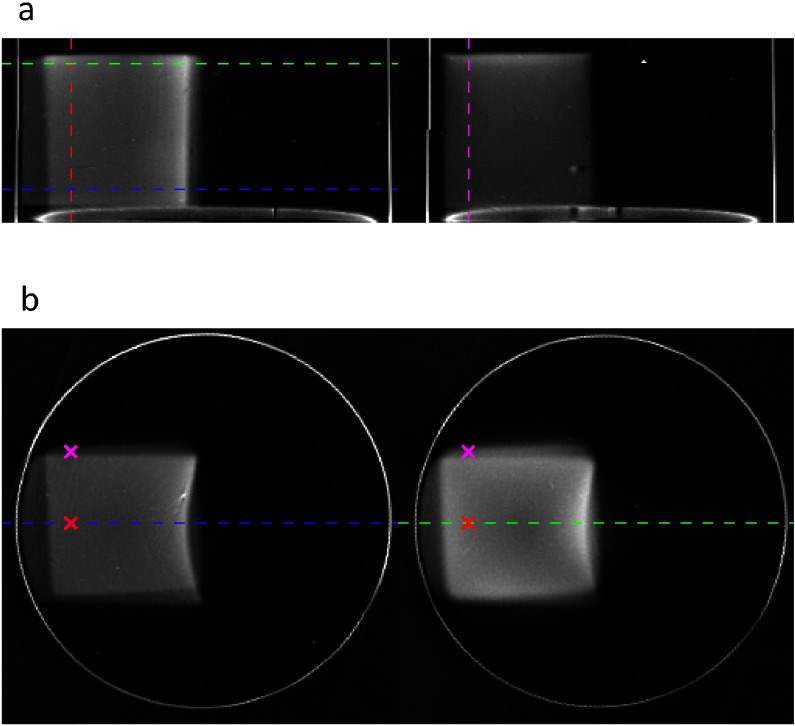
Result of the experiment to irradiate sample G with two overlapping Bragg peaks along perpendicular axes. (*a*) Coronal *xz*, and (*b*) transverse *xy* slices through the sample. The dotted lines and crosses correspond to the positions of the profiles displayed in figure 6.

**Figure 6.**
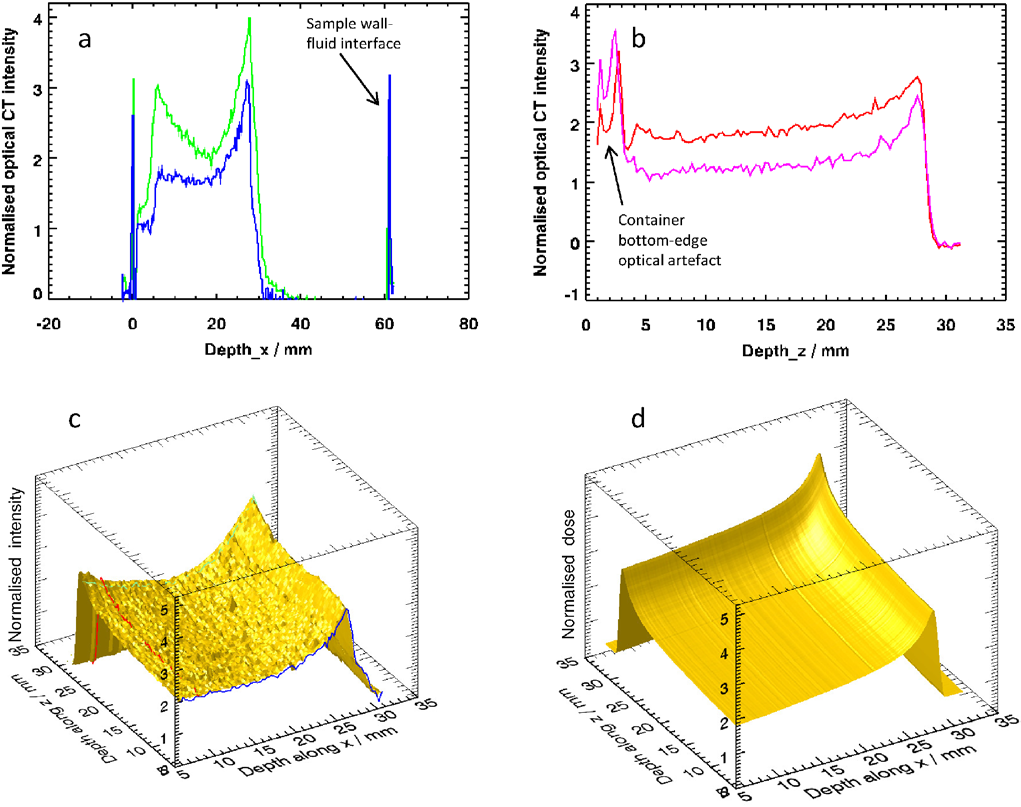
Result of the experiment to irradiate sample G with two overlapping Bragg peaks along perpendicular axes: (*a*) profiles along the *x*-direction at the locations shown by the blue and green dotted lines in figure 5; (*b*) profiles along the *z*-direction at the locations represented by the dotted lines and crosses in figure 5; (c) 2D view of the data from the plane through the sample represented by the green dotted line in figure 5(*b*), with the profiles from (*a*) and (*b*) superimposed; (*d*) corresponding dose prediction based on equation (5).

Figure 5 displays the results obtained by irradiating sample G with an entrance dose of 4.0 Gy from one end, followed by the same dose from the side. Two different planes are shown for each of the coronal and transverse sections. Note the curved Bragg peak and the overlaying of the two dose contributions, which is particularly evident in figure 5(*b*).The profiles corresponding to the dotted lines and crosses in figure 5 are shown in figures 6(*a*) and (*b*), whilst in figure 6(*c*), we show the 2D distribution of measured image intensity as a function of both depth along the *x*-axis (across the sample) and depth along *z* (along the length of the sample). Figure 6(*d*) is again based on equation (5)

## 5. Discussion

The overall aim of this work is to examine whether it is feasible to use quantitative 3D imaging of PRESAGE^®^ radiosensitive samples, with the current technology, for proton dosimetry. This breaks down into four separate questions:

• Are the existing samples suitable?
• Is the existing readout technology suitable?
• Is our theoretical understanding of the processes involved sufficient?
• Are any of the features that are observed here under extreme conditions relevant for more typical dose distributions proposed for the clinic?

The profiles of figures 2(*b*) and (*c*) show that the measured quenching effect becomes more pronounced with proximity to the Bragg peak (i.e. with increasing LET), as might be expected. This has been shown by previous authors. The first new aspect of this work is to demonstrate how important correct normalization of the data is. A cursory glance at figure 2(*c*) might suggest that quenching is strongly dose-dependent. However, figure 3(*a*) gives us more insight into what is happening. Although the curve fitted is empirical—we are not at this stage proposing a full theoretical model for the process—it seems clear that the dosimeter response is exponential. Non-linear effects were not observed by (Zhao *et al* 2012) for the same PRESAGE^®^ formulation, but their experiments considered a maximum dose of only 10 Gy. Previous work by Al-Nowais *et al* using a different formulation of PRESAGE^®^ also observed an exponential response, but with a much higher *D*′ value of 1070 Gy (Al-Nowais *et al* 2010).

The green curve of figure 3(*a*) is our current best estimate of the quantity $~I(D,~{{\mathcal{L}}_{0}})~$ from equation (1), whilst the blue curve is $I\left(D,~\mathcal{L}\right)$ at the Bragg peak. The difference in image intensity corresponding to the quenching effect is thus represented by the red arrow. Although the errors on the points in figure 3(*a*) are relatively small, the extrapolated portion of the curve is not well estimated, because the acquired data do not extend to high enough doses.

Taken together figures 3(*a*) and (*b*) demonstrate that both the non-linear response and quenching are important for entrance doses above 4 Gy and if this effect is ignored and a single calibration value is used to correct the quenching effect, then significant errors may be introduced into the final measure of dose.

The results for the 2 and 4 Gy samples are very similar (*R* = 0.63), but the ratio to the ion chamber reading is 25% lower (*R* = 0.46) at the Bragg peak of the 14.7 Gy sample D. Interestingly, the analysis reported in figures 2(*d*) and 3(*a*) show that it is the LET-independent non-linear response of the PRESAGE^®^ that contributes most to this reduction. An entrance dose of 14.7 Gy corresponds to a true physical dose of approximately 66 Gy, which is extremely large compared with what would be delivered in the clinic. Figure 2(*d*) shows that the quenching function is slowly varying and, in principle, we can thus predict the quenching profile for other entrance doses.

As described in the Theory section above, the existence of an LET-dependent quenching function means that a *direct back-calculation from optical CT image intensity to measured dose is not possible*. Our goal is thus to establish whether a *forward* model is possible: can we predict the image intensity if we know both the expected dose and the expected LET at which this dose is deposited? The second experiment is designed to the provide first steps to answering this question. Samples E and F provide two simple case studies with a view to creating a forward model based on the data measured in the first experiment. Sample G is an attempt to measure empirically the joint quenching function for all combinations of two LET values. By arranging for two Bragg peaks to deposit dose in orthogonal directions, each pixel on the coronal plane defined by the green dotted line in figure 5(*b*) is irradiated with a different combination of two depth (and hence LET) values. In principle, even if equations (5) and (6) were not valid, (i.e. the results of the two irradiations could not be treated separately), these data might give enough information to create an appropriate forward model.

The results of the second experiment raise a number of interesting questions regarding both the dosimeter performance and the suitability of the current optical CT readout apparatus for reading out doses.

• *Sample stability and image normalization*: The 4 Gy sample B from the first experiment and samples E and F from the second experiment were scanned on three consecutive days. All the data were normalized by dividing by the same value to give the results shown in figures 4(*a*) and (*b*). Comparing the Bragg peak of the singly-irradiated region (distal to the Perspex-shifted Bragg peak), we see an increase in optical density of 2% for sample E over sample B and an increase of 10% for sample F over sample B. These effects may be due to continuing reactions within the PRESAGE^®^, since all of the samples A–G darkened very significantly during the weeks following these scans. The effect is known to depend on the PRESAGE^®^ formulation used and there are also suggestions of a dose-dependence to the darkening (Skyt *et al* 2012).
• *Interpretation of the mixed-LET region*: The purple dotted lines in figure 4 show the results of assuming a linear dose–response model for PRESAGE^®^. Here, equation (5) was applied, but with a dose-dependent attenuation function. The maximum physical dose received by the sample was approximately 20 Gy, which is sufficiently large for the non-linear dose–response of the dosimeter to become evident. It is thus not surprising that the model is inadequate to describe the proximal data in figure 4(*a*). (Note that the proximal data are well described in figure 4(*b*), but the second Bragg peak does not match. Were the data to be normalized here, the results would be as in 4a.)

The green lines in figure 4 show the results of a model based on equation (6). We chose the simplest model consistent with the data, which was a linear variation of *q* with depth. The match is surprisingly good, but further work is necessary to investigate different models and provide a theoretically underpinning for this empirical result.

• *Scanner performance for large high-dose areas*: The results of figures 5 and 6 are indicative of limitations in the performance of the optical CT scanner when imaging large high-dose areas within a single slice. This is particularly evident in figure 5(*b*). The left-hand slice, taken near the entrance region of the end-on beam, shows the expected uniformity within the majority of the irradiated square. The true physical dose here is around 8 Gy at the left, rising steeply to a Bragg peak of true dose around 22 Gy at the right of the image. No significant variation is expected or seen across the central square in the *y*-direction. By contrast, the right-hand slice is taken from the Bragg peak region of the end-on irradiation. The contribution from this beam is around 18 Gy throughout the slice. The bright region at the right corresponds to a physical dose of around 36 Gy where the two Bragg peaks superimpose. In this slice, we see unexpected variations along vertical profiles, with the centre of the square having a lower image intensity than predicted. This manifests itself in the strongly dipped green profile in figure 6(*a*), which does not correspond to the simulations and which we do not believe relates to dosimeter quenching or non-linear dosimeter response. Further investigations into the cause of this effect are needed, but we note that previous work by other authors has reported discrepancies at the centre of large irradiated regions—see figure 7 of (Islam *et al* 2003). However, as discussed below, although these scanner-related limitations for probing extreme conditions hamper the investigation of the physical mechanisms occurring in the dosimeter, they are unlikely to restrict the use of optical CT in the typical clinical regime.

Regarding the question of relevance to realistic treatments, we note that our experiments were designed to address the most challenging cases, with extremely high dose and strong influence of LET. These situations correspond to the ‘poorest’ performance of both the chemical dosimeter and the optical scanner. However, the similarity of the quench profiles for doses 2.0 and 4.0 Gy (figure 2(*b*)), together with the observed degree of curvature in figure 3(*a*) provide grounds for cautious optimism. If the maximum total dose is restricted to around 18 Gy, the equivalent of a single fraction with entrance dose of 4 Gy, then equation (3) with a linear model for *α*, might be sufficent to model correctly image intensities in PRESAGE^®^ and so apply the ‘forward model’ approach described.

Unfortunately, there is a paucity of reported data for proton irradiations other than the case of a pure Bragg peak. In the two such studies that have been performed (Zeidan *et al* 2010, Zhao *et al* 2012), the regions that failed gamma analysis were primarily at the distal ends of modulated beams (spread-out Bragg peak). These regions are the ones with the least admixture of LET values and thus precisely the places where one might have expected to see the most obvious manifestations of quenching. However, in both cases, the authors found the experimentally measured signal to be *higher* than that calculated (Zeidan figure 4, top right; Zhao figure 9). This effect cannot easily be explained in terms of quenching or non-linear dosimeter response. Zeidan *et al* put forward the hypothesis of a genuine difference in physical dose delivery between the ion chamber and gel measurements, caused by variation in the accelerator beam current. Needless to say, the lack of a reliable reference in such cases complicates the situation still further.

## 6. Conclusions

We have laid out a number of problems inherent in performing proton dosimetry using 3D imaging of chemical dosimeters. The exemplar used here was optical computed tomography of the radiochromic plastic PRESAGE^®^, but similar results will apply to a number of other combinations of dosimeter and imaging readout. The primary issue arises from a variation with proton LET of the dose-sensitivity of the optical response, which is also known as the ‘quenching’ effect. Simple theoretical arguments lead to the conclusion that the total absorbed dose from multiple irradiations with different LETs cannot be uniquely determined from post-irradiation imaging measurements on the dosimeter. Thus, a direct inversion of the imaging data is not possible. A second problem is the potential for a non-linear response of the dosimeter at high doses, something which may or may not be independent of LET. Our experimental work investigated the feasibility of a solution involving a ‘forward model’ to predict image intensity followed by comparison with experimental measurements of optical density. In order to implement this practically, one would need to extend the concept of a ‘treatment plan’, to include both the dose deposited at each point and the LET value.

Dosimeter response as a function of depth (and hence LET) and dose were measured. For peak physical doses of less than around 20 Gy, the assumption of a linear dosimeter response was found to be valid for our particular PRESAGE formulation, but for higher doses, there was a marked decrease in optical response at the Bragg peak (an extra 25% at an entrance dose of 14.7 Gy). This was found to be primarily due to the non-linear response of the dosimeter, rather than an enhanced quenching effect.

A second experiment, involving samples irradiated with two separate beams, concluded that a linear model with separate calculation of the quenching for multiple irradiations followed by addition of the corresponding image intensities did not yield the measured result where two beams overlapped. A second model, which used the concept of an ‘effective dose’ matched the experimental results more closely. An attempt was made to measure directly the quench function for two proton beams as a function of all four variables of interest (two physical doses and two LET values). However, this approach was not successful because of limitations in the response of the scanner.

It is clear that 3D proton dosimetry is not yet a ‘turn-key’ activity. Nevertheless, the results here should not be taken as an indication that it will not work. The experiments were deliberately designed to probe extreme situations, in terms of beam energy, irradiation pattern and dose delivered, whereas typical clinical dose distributions will be more moderate. There is thus a need for a future programme of research to understand better the underlying physico-chemical phenomena occurring in the dosimeter, to characterize the response of the optical CT scanner at high doses and to determine an appropriate regime in which such measurements can used successfully for dose verification.
